# Bilobed testicle diagnosed by ultrasound in a 9-year-old boy

**DOI:** 10.1016/j.radcr.2022.09.059

**Published:** 2022-10-31

**Authors:** Romaissaa Boutachali, Sakina Nahmed, Othmane Jbara, Dalal Laoudiyi, Kamilia Chbani, Siham Salam

**Affiliations:** Department of Pediatric Radiology, Ibn Rochd University Hospital, Faculty of Medicine and Pharmacy of Casablanca, 1, quartiers des hôpitaux, 20100, Casablanca, Morocco

**Keywords:** Bilobed testicle, Scrotal mass, Incomplete polyorchidism

## Abstract

Bilobed testis is an extremely rare congenital malformation, and even rarer on the right side. Only 7 cases have been reported in the literature. We describe the case of a 9-year-old boy with a right bilobed testicle confirmed on ultrasound and discovered incidentally as a mass on physical examination. The aim of our work is to consider the bilobed testicle as a differential diagnosis of a testicular mass despite its rarity and to show the importance of ultrasound and MRI for its definitive diagnosis to avoid unnecessary surgery.

## Introduction

Bilobed testis is an extremely rare congenital malformation in children, more common on the left side [1,2]. Although the etiology is still unknown, it is speculated that this malformation may be an incomplete expression of polyorchidism with less than 200 cases reported [1,3]. It is overall a benign condition according to previous studies [3,4], but it may lead to a higher risk of torsion or even malignancy [4]. Its treatment is so far conservative, but it is difficult to evaluate the long-term development of the disease due to its rarity [4]. Only 7 cases have been reported in the literature to date [4].

We describe here a case of right bilobed testis in a 9-year-old boy confirmed by scrotal ultrasound.

## Case report

A 9-year-old boy with no particular personal or family history was referred to the department of pediatric surgery and radiology for a right scrotal painless mass, not increasing in volume, with no inflammatory signs. It has appeared 2 years prior to his admission.

The clinical examination revealed a round, painless, mobile mass, well-limited, in the upper pole of the right testicle. The rest of the physical examination didn't reveal anything particular.

**Fig. 1:**
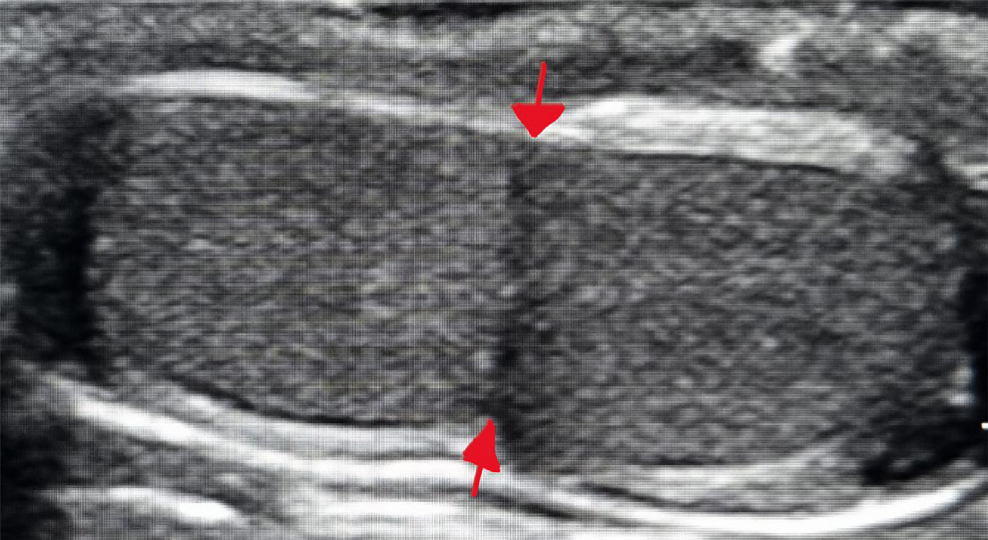
Transverse ultrasound showing a right bilobed testicle (red arrow shows the separation of the 2 lobes).

Faced with this clinical picture, a scrotal ultrasound was performed revealing a hypoechoic mass attached to the upper pole of the right testicle with normal vascularization shown in the Doppler ultrasonography measuring 10 × 6.5 × 5 millimeters. The mass had a similar size and echotexture with normal adjacent testis (Fig. 1). The left testicle was of a similar size, measuring 21×14×10 millimeters with normal vascularity and normal‑appearing of both epididymis and spermatic cords.

Because of the echogenicity corresponding to a testicular structure and the absence of complications such as testicular torsion, the diagnosis of bilobed testicle was retained.

Conservative treatment was indicated with quarterly ultrasound monitoring of the patient.

## Discussion

Bilobed testis is an extremely rare testicular malformation [1], several researches have been made to explain its mechanism, but at the moment no theory can be confirmed [1]. Though we can assume that it is an incomplete form of polyorchidism [3], it may result from incomplete division of the genital ridge by the peritoneal band [4]. It is much rarer and more interesting to study [2]. However, the main distinguishing feature between these 2 diseases in the case of polyorchidism, is that the supernumerary testicle is smaller than the main testicle [3]. It is usually asymptomatic [5]. It has been associated with several pathologies such as malignancy, inguinal hernia and testicular torsion [3].

According to previous studies the bilobed testicle is considered a benign entity, however a debate still persists over the risk of torsion and malignancy on bilobed testicle cases [3,4].

Historically, the definitive diagnosis of polyorchidism was made by surgical exploration, nowadays ultrasound and MRI are sufficient to diagnose polyorchidism including bilobed testicle.

The management of polyorchidism still remains controversial [1,4]. Some authors recommend conservative treatment [1] while others have recommended surgical management including partial orchiectomy or orchidopexy depending on the complication, based on the suspicion of malignancy and the high prevalence of testicular torsion [5].

As mentioned above in the case report, in our experience, given the absence of complications, surgical exploration was not necessary.

## Patient consent

Written and informant consent for publication of the case was obtained from the patient.
